# Morphological changes and protein degradation during the decomposition process of pig cadavers placed outdoors or in tents—a pilot study

**DOI:** 10.1007/s12024-023-00632-3

**Published:** 2023-05-01

**Authors:** J. Geissenberger, J. Amendt, J. Klampfer, L. Thuemmel, L. Jakob, F. C. Monticelli, P. Steinbacher, S. Pittner

**Affiliations:** 1 Department of Environment and Biodiversity, University of Salzburg, Hellbrunner Straße 34, 5020 Salzburg, Austria; 2 Institute of Legal Medicine, Goethe-University, Frankfurt, Frankfurt, Germany; 3 Department of Legal Medicine and Forensic Neuropsychiatry, University of Salzburg, Salzburg, Austria

**Keywords:** PMI estimation, Protein degradation, Field study, Muscle, Pig, Concealment

## Abstract

The delimitation of the postmortem interval (PMI) is of utmost importance in forensic science. It is especially difficult to determine the PMI in advanced decomposition stages and/or when dead bodies are found under uncommon circumstances, such as tents, or other (semi-) enclosed environments. In such cases, especially when insect access is restricted, morphological assessment of body decomposition is one of the remaining approaches for delimitation of the PMI. However, as this method allows only vague statements/indications about the PMI, it is required to develop new and more reliable methods. One of the most important candidates is the biochemical analysis of protein degradation. In this regard, it has been demonstrated that specific skeletal muscle protein degradation patterns characterize certain time points postmortem and thus can be used as markers for PMI estimation. In order to test this method in different micro-environments, a pilot study using ten pig carcasses was conducted in summer in Northern Germany. The cadavers were openly placed outside (freely accessible for insects), as well as enclosed in tents nearby, and left to decompose to investigate decomposition processes over a time course of 10 days. Muscle samples of the *M. biceps femoris* were collected on a regular basis and processed via SDS-PAGE and degradation patterns of selected proteins identified by Western blotting. In addition, morphological changes of the cadavers during decomposition were assessed using the total body score (TBS). Results showed that postmortem protein degradation patterns are largely consistent between treatment groups (open field versus tents) despite major morphological differences in the decomposition rate. This field study provides evidence that muscle protein degradation is mostly unaffected by different levels of exposure, making it a sufficient candidate for PMI delimitation under various circumstances.

**Supplementary Information:**

The online version contains supplementary material available at 10.1007/s12024-023-00632-3.

## Introduction

The delimitation of the postmortem interval (PMI) is a major task and a continuous challenge in forensic routine. It is particularly difficult when corpses are found after a considerable amount of time after death, due to the limited number and validity of available methods. In such cases, important methods for PMI determination are the analysis of necrophagous insects colonizing the dead body, and additionally the quantification of the degree of body degradation (via morphological assessment). Particularly the colonization by fly maggots and beetle larvae has a considerable impact on the decomposition process of dead bodies [1–4]. PMI determination becomes even more complicated when corpses are found under uncommon or unusual circumstances, e.g., where there is limited access of the necrophagous insects to the bodies and/or under specific environmental conditions [5–8]. A limited access to the cadavers leads to altered species compositions and decreased numbers of colonizing insects [4, 9–13]. Specifically, the concealment of dismembered bodies in boxes or plastic waste sacks considerably decreases the rate of decomposition [1, 11]. Besides the reduction of insect colonization, other factors such as humidity, the microbiome, and metabolic processes may add to this difference.

In general, it is important to increase the portfolio of methods that can be used for PMI delimitation in order to get the most reliable and precise outcome. In that respect, a promising addition is biochemical analysis of postmortem muscle protein degradation patterns. This method identifies specific degradation events of certain proteins, which provide reasonable markers for PMI delimitation [14–16]. In addition, the protein decomposition process is often accompanied by the occurrence of degradation products at specific time points, which can also be used for PMI determination. Previous studies showed highly predictable and temperature-dependent results of protein degradations patterns under standardized conditions [14, 17, 18]. In addition, a recent field study showed that postmortem muscle protein degradation in pigs was (largely) unaffected by varying exposure conditions (sunlight/shadow, direct/indirect coverage, etc.) [19]. This method is also valuable due to the fact that muscle proteins degrade at different time periods after death [15], allowing delimitation of early as well as late PMIs.

However, considerable research is yet required to increase our understanding of how protein decomposition is influenced by individual and environmental factors in order to provide a broad applicability of this method. In general, autolysis and putrefaction characterize the process of soft tissue degradation. During decomposition, endogenous cells are destroyed by enzymes produced by the organism itself (autolysis), whereas external bacteria and fungi digest the tissue by splitting proteins under anaerobic conditions (putrefaction) [20]. Currently it is unknown whether the extreme conditions in sealed bags also have an influence on autolysis, putrefaction, and protein degradation dynamics. Moistening (as well as drying) of the skin of a body may affect the underlying muscle and therefore the preservation or degradation of soft tissue and muscle proteins [21]. This may be crucial for late PMI stages and mummified corpses since there is a lack of reliable methods applicable for time since death estimates in these stages [22, 23].

The present comparative study aims to provide new insights on the decomposition process and protein degradation in cadavers placed outside or inside of tents. The field study was conducted using five pig carcasses, left to decompose under natural environmental conditions at an open forest area, as well as five pig cadavers placed inside of tents at the same site. Morphological changes of the cadavers during decomposition were assessed using the total body score (TBS). Different scoring methods were established over the last years to quantify the degree of body decomposition of human remains [24–26] and different animal cadavers [27, 28] as a valuable addition to other methods for PMI estimation [6, 19].

In addition to TBS evaluation, muscle samples of the *M. biceps femoris* were collected, processed via SDS-PAGE and degradation patterns of specific proteins were identified by Western blotting. Proteins were selected according to their previously characterized differences in degradation rates [17, 19, 29] with degradation events occurring either rather early (within one or two days) or in later postmortem phases (several days). Results are thought to improve our understanding of postmortem body and protein degradation under natural environmental conditions compared to sealed (micro-)environments.

## Material and methods

### Animals and experimental setup

Ten sub-adult pigs (commercial crossbreed animals, 12–13 weeks old, 30 ± 3 kg) were used for this field study conducted during the summer period of June 2021. The animals were killed in accordance with all international ethical requirements for the use of animals in experimental research studies, by using a mixture of deep anesthetics (0.5 mL Stresnil and 10 mL 10% ketamine per 10 kg of body weight) and T61—a mixture of tetracaine hydrochloride, mebezoniumiodid, and embutramide. Subsequently the carcasses were transported to a forensic research outdoor station in Northern Germany (N 51.915, E 7.907) and randomly allocated to two treatment groups (*n* = 5 per group)—either located at an open glade under moderately high trees (mainly oaks and hornbeams) or individually placed in closed tents in the same forest area. Open-field pigs were covered with a wire mesh to prevent vertebrate scavenging and were left to decompose under natural conditions. Additionally, all pigs were equipped with rectal sensors, measuring temperatures hourly. Furthermore, ambient temperatures were documented using data loggers in near proximity to the openly placed cadavers and centrally mounted on the ceiling inside the tents. The sample collection and evaluation of the decomposition progress and morphological changes were evaluated daily for open-field pigs. In order to mimic and preserve a closed-off environment as good as possible, the tent pigs were sampled and TBS evaluated only every 5 days.

### Morphological assessment

To quantify the degree of decay, the evaluation of the total body score (TBS) is a commonly used tool. In this study, morphological scoring was performed according to Keough et al. [28] which was specifically designed to evaluate postmortem changes of pig cadavers. Morphological changes were assessed over a time period of 25 dpm for open-field pigs and of 45 dpm for tent pigs.

In order to reduce observer bias, evaluations were independently assessed by at least two observers and discussed prior to documentation. The evaluation sheet uses a scoring system, which subdivides the cadaver in three different regions: (i) head, (ii) trunk, and (iii) limbs. These body regions were individually evaluated for each pig (*n* = 10) and morphological observations were scored to a maximum of (i) 13, (ii) 12, and (iii) 10 scoring points. The total body score was obtained by adding the three values. Ultimately, mean values and standard deviations were calculated for each treatment group (open field and tent). Additional observations during TBS evaluation, not mentioned in the scoring sheets, were documented individually.

### Muscle sampling and protein analysis

Muscle samples were collected daily from each open-field pig (*n* = 5) for 7 dpm and every 5 days for each pig placed in a tent (*n* = 5) until 10 dpm. Samples from the *M. biceps femoris* were taken via biopsy and individually stored in 1 mL RIPA buffer (SIGMA) containing a protease inhibitor cocktail (ROCHE) in a cooled styrofoam box at the research outdoor station and later at − 20 °C until further use.

Sample processing, protein analysis, and statistical evaluation were carried out according to a standardized procedure [29] with minor adaptations.

Details are described in Supplementary File S1.

## Results

### Temperature

The field study was conducted during a summer period with ambient temperatures above average for (Northern) German conditions. However, temperatures displayed a typical day-night cycle with higher values at daytime and lower temperatures during the night. In detail, temperature measurements revealed a mean ambient temperature of approx. 18.6 °C. Average minimum and maximum values were 15.7 °C and 21.7 °C, respectively. The temperatures within tents displayed a mean of 19.4 °C, with minimum and maximum mean values of 15.2 °C and 25.1 °C, respectively.

**Fig. 1:**
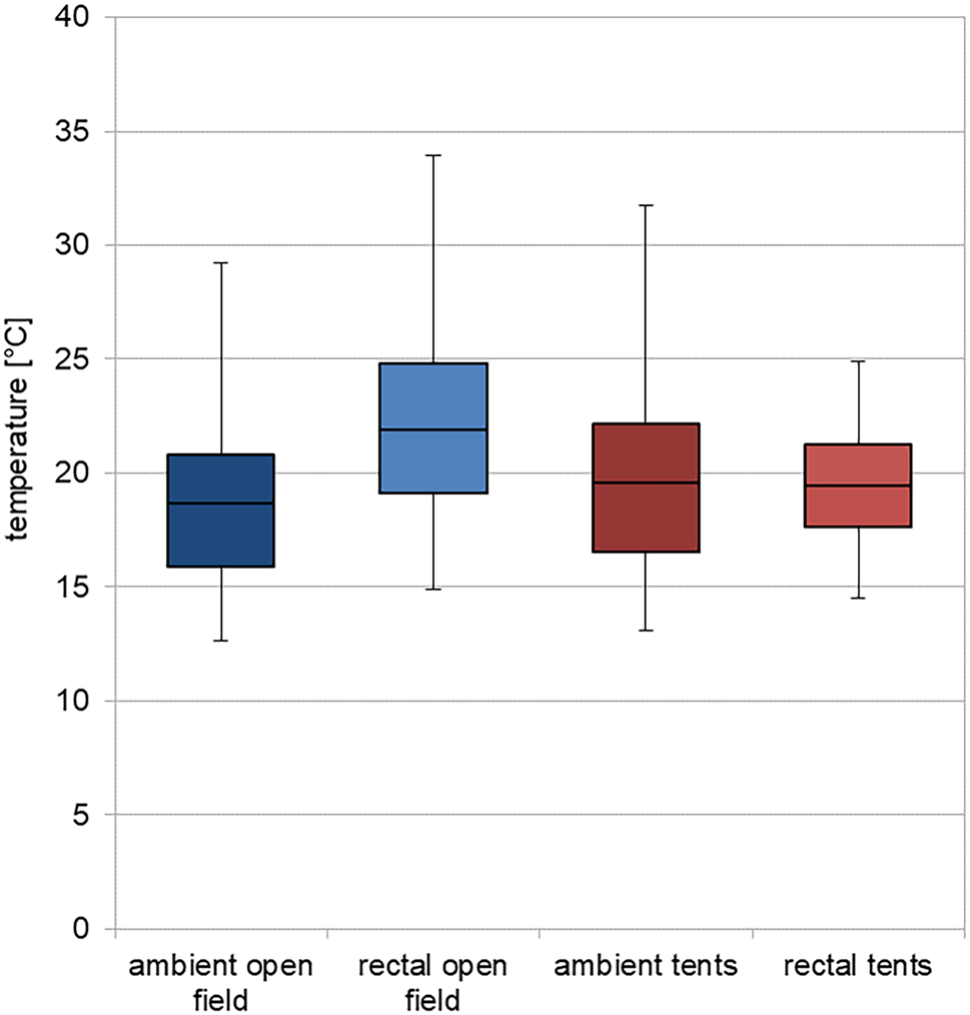
Means of ambient (dark color) and rectal temperatures (light color) of open-field pigs (blue) and tents [46], including standard deviations. All mean temperature values vary between 15 and 25 °C. Rectal (body core) temperatures are higher in relation to respective ambient temperatures, especially rectal temperatures of open field cadavers

Body core (rectal) temperatures of all carcasses (*n* = 10) showed fluctuating temperatures in a circadian rhythm. They were slightly higher than ambient temperatures, especially during the first 2 days of the experiment. In addition, from 4 dpm onwards, open-field pigs displayed even higher rectal temperatures compared to environmental temperatures, most probably due to the heat produced by fly maggots, extensively colonizing the corpses (see below). Mean rectal temperature of the openly placed pigs was 25.3 °C during the experiment. Minimum and maximum rectal temperatures of open-field pigs were 18.7 °C and 24.9 °C, respectively. Tent pigs, by comparison, showed slightly lower temperatures than openly placed pigs with mean values of 19.7 °C and minimum and maximum temperatures of 17.2 °C and 23.3 °C, respectively (c.f. Fig. 1 and Supplementary Fig. S1).

### Morphological assessment

**Fig. 2:**
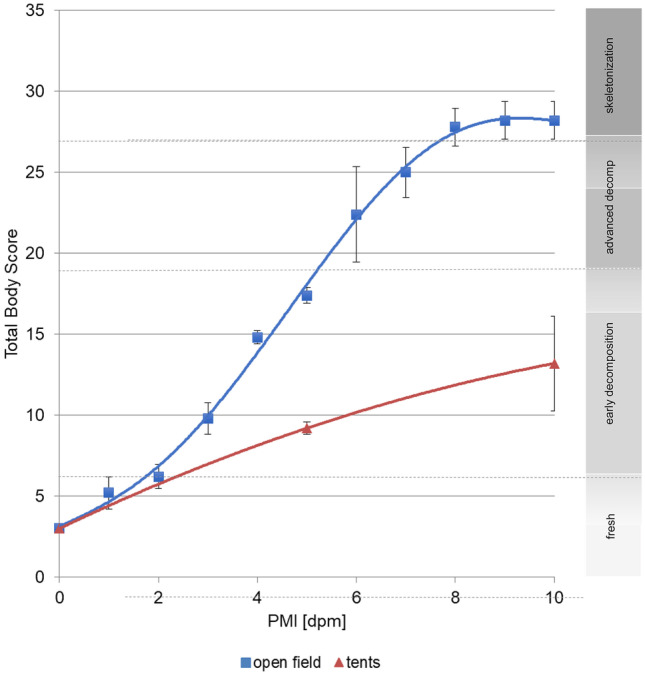
Development of total body score (TBS) mean values of open-field pigs (blue squares) and tent pigs (red triangles), including standard deviations and trend lines. Both treatment groups depicted an increase of TBS values over the investigated time course of 10 dpm for both treatment groups. TBS values of open-field pigs are significantly higher than tent pigs

At the onset of the experiment, neither *rigor*, nor *livor mortis* was detectable in any of the animals. However, after that, significant differences were detected (Fig. 2).

#### Open-field pigs

**Fig. 3:**
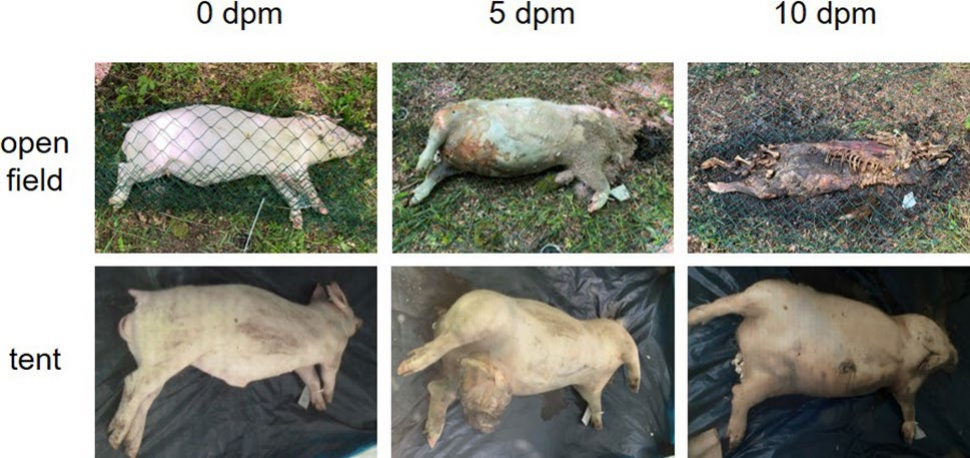
Representative progression of morphological changes during the decomposition of open-field pigs (top row) and tent pigs (bottom row) over the time course of 10 dpm

All open-field cadavers decomposed within only few days and went from a fresh stage to advanced decomposition very quickly (Fig. 2a). Total body scores developed from 3 at 0 dpm to 29 points within only 10 days (Fig. 3).

In detail, at the beginning of the experiment (0 dpm), all five pigs were in a fresh state. Shortly after death (approx. 2 h), adult flies started oviposition on the cadavers. Within the first 5 days of the experiment (5 dpm), all pigs showed typical signs of early decomposition, including extensive maggot and adult beetle colonization of head and trunk, green to black discoloration all over the cadaver. In addition, limbs and the abdominal of the cadavers were characterized by a marbling and maximum bloating, as well as extensive skin slippage.

Between 5 and 10 dpm, all open-field cadavers entered advanced decomposition stages, which was mainly characterized by extensive maggot colonization all over the cadaver, the beginning mummification of trunk and limbs, including leathery appearance of the skin and bone exposure. The trunks displayed dried brown areas with skin slippage and extensive hair loss. At 10 dpm, a complete bone exposure of the head and limbs was detectable and marked the onset of the skeletonization stage. In addition, some of the cadavers showed exposed bone in the abdominal region (mainly rib cage).

For a detailed description of morphological changes in the open-field pigs, see Supplementary File S1.

#### Tent pigs

In general, the decomposition process of the pig cadavers placed inside tents was much slower than in the open-field pigs (Fig. 2b). Four of five pigs showed similar morphological changes at distinct PMI phases with total body scores from 3 at 0 dpm up to a maximum of 12 within 10 dpm. One pig showed an exceptionally advanced decomposition rate compared to the remaining cadavers with a TBS of 19 at 10 dpm (Fig. 3).

At the beginning of the experiment (0 dpm), all pigs were in a fresh state. At 5 dpm, tents were opened for the first time and revealed similar initial morphological changes amongst all cadavers, aligned with signs of early decomposition. These signs included green discoloration and bloating of the cadavers with maggot colonization (mainly in the mouth region) and marbling of the skin.

At 10 dpm, four pigs displayed a similar putrefaction progress, still typical for an early decomposition stage. Most prominent signs were bloating of head, neck, and limbs, post-bloated trunks, purging of decomposition fluids, skin slippage, and a green-to-black discoloration of the cadavers. One of the cadavers showed exceptional morphological changes and signs of an advanced decomposition phase. These changes included extensive skin slippage on neck and limbs, maggot colonization, and migration of post feeding larvae to the tent corners. In addition, a moist decomposition with foam building around the cadaver and bone exposure of trunk and limbs was detectable.

For a detailed description, see Supplementary File S2.

### Postmortem degradation of muscle proteins

Due to the high temperatures and substantial insect activity, decomposition has progressed in a manner that muscle tissue collection was substantially hindered already after day 7 in the open-field pigs, whereas muscle samples from pigs in a sealed environment (tents) could still be obtained on day 10 postmortem in all animals. Results showed that investigated muscle proteins degraded in a similar fashion, regardless of exposure of the animals to the environment (open field or tent) and despite differences in the decomposition process and morphological changes amongst treatment groups. Even under extreme ambient temperatures, some proteins remained stable over the investigated time, while others displayed a complete degradation of the native form, in part accompanied by the occurrence of distinct degradation products at defined PMI phases.

#### Open-field pigs

**Fig. 4:**
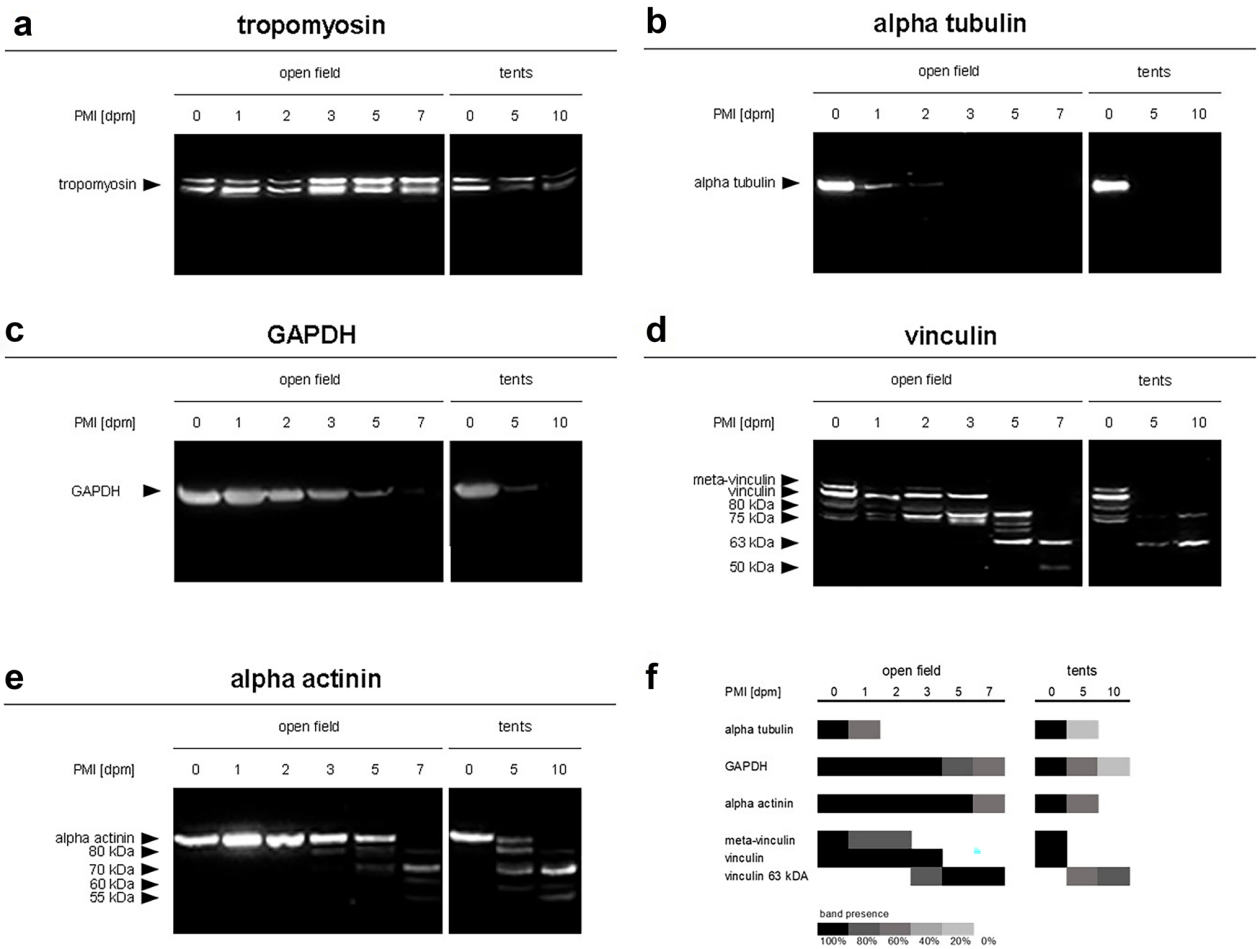
Results of the analysis of muscle protein degradation. **a–e** Representative Western blot results depicting muscle protein degradation patterns of tropomyosin (**a**), alpha tubulin (**b**), GAPDH (**c**), vinculin (**d**), and alpha actinin (**e**), over a time course of 7 dpm for open-field pigs, and over 10 dpm for tent pigs. **f** Heatmap of selected native proteins and degradation products, depicting the abundance of protein bands over the investigated time course. Native forms of alpha tubulin, GAPDH, vinculin and alpha actinin, as well as meta-vinculin showed a decrease of band intensity, the vinculin split product of 63 kDa (**e**) depicted an increase of band presence

Tropomyosin with its native double-band appearance remained stable over the time course of this field study in all open-field cadavers. The protein showed no signs of degradation, nor displayed any degradation products over the investigated time (Fig. 4A).

**Fig. 5:**
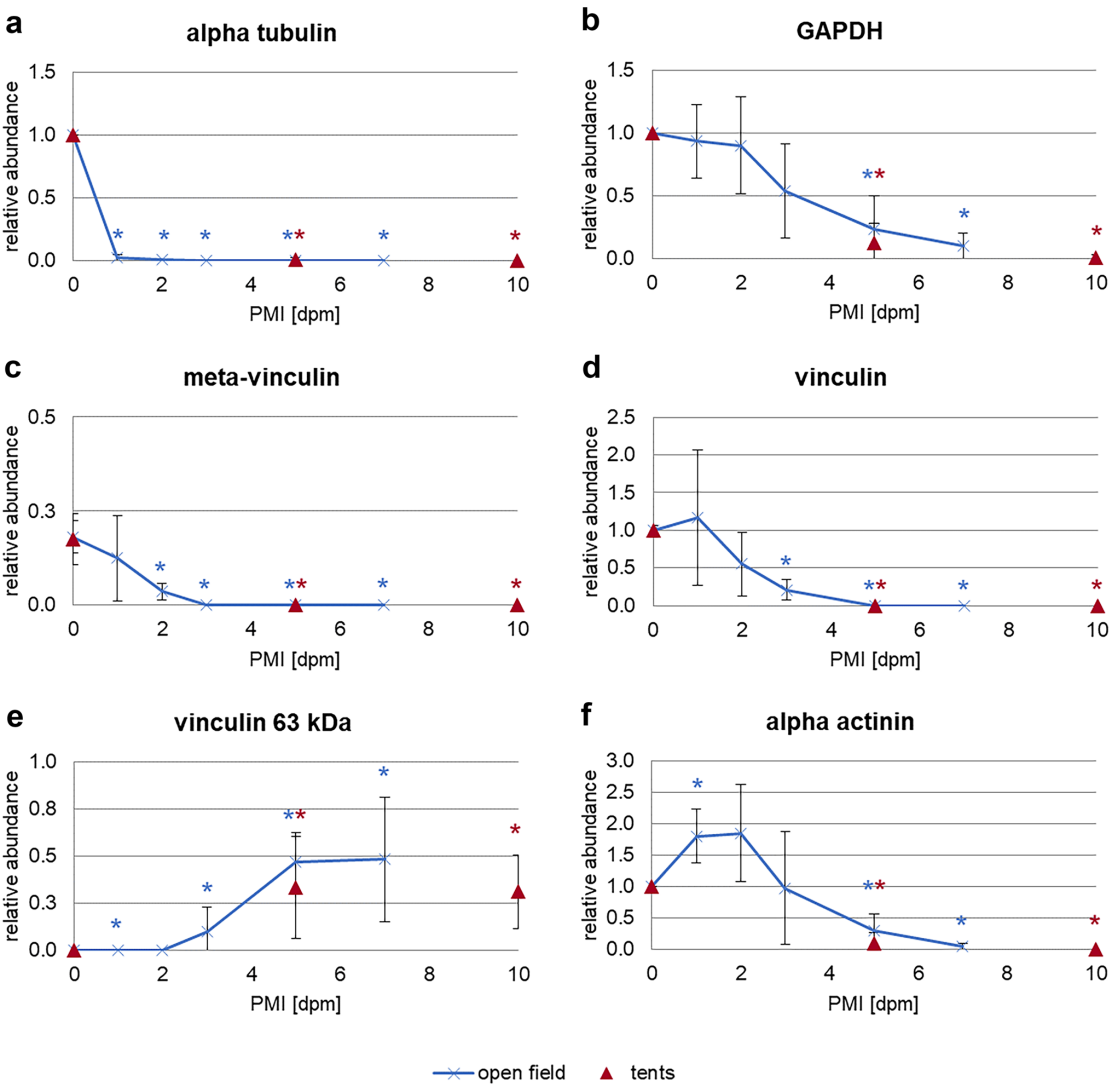
Relative abundances of alpha tubulin (**a**), GAPDH (**b**), meta-vinculin (**c**), vinculin (**d**), vinculin degradation product 63 kDA (**e**), and alpha actinin (**f**), depicting the abundance of protein bands over the investigated time course of 7 and 10 dpm, respectively, for open-field pigs (blue line) and tent pigs (red triangle). Standard deviations are included and significances (*p* < 0.05) are depicted by asterisk in blue for open-field pigs and in red for tent pigs. All native proteins (**a**, **b**, **d**, **f**) as well as meta-vinculin (**c**) showed a decrease in relative abundance in both open-field and tent pigs. The vinculin degradation product of 63 kDa (**e**) depicted an increase in relative abundance over the investigated time in both treatment groups

In comparison, alpha tubulin and GAPDH fully degraded without the formation of any split products. In detail, the native band of alpha tubulin disappeared after 1 dpm in all open-field pigs (Figs. 4B and 5A). GAPDH fully degraded in 2 of 5 open-field animals over the investigated time (Figs. 4C and 5B), others displayed a fading in band intensity of the native protein.

The proteins vinculin and alpha actinin also exhibited a complete degradation of the native band over time in open field. This degradation was accompanied by the appearance of degradation products at distinct time points (Fig. 4D + E). Analysis showed that in openly placed animals, native vinculin was fully degraded after 3 dpm (Fig. 5D), and native alpha actinin completely disappeared at 7 dpm (Fig. 5F). Split products of alpha actinin with molecular weights between 80 and 55 kDa occurred from 3 dpm onwards but displayed no statistical regularity in their appearance, whereas some splice variants and degradation products of vinculin appeared at specific time points with high probability. Specifically, the vinculin splice variant with a molecular weight of 135 kDa, often referred to as meta-vinculin, degraded within the first 3 days postmortem (Fig. 5C). In addition, the vinculin protein band with a molecular weight of approx. 63 kDa significantly appeared from 3 dpm onward (Fig. 5E).

#### Tent pigs

Like in open-field pigs, native tropomyosin remained stable over the investigated time course in all tent pigs without any signs of degradation (Fig. 4A).

Western blot results revealed significant loss of the native alpha-tubulin band in all tent carcasses on sampling day 5 dpm (*p* < 0.05), indicating a full degradation of the protein within the first 5 days postmortem (Figs. 4B and 5A).

Similar to open-field pigs, 4 of 5 tent pigs showed a complete degradation of native GAPDH at 10 dpm (Figs. 4C and 5B). The remaining pig showed a stable native protein band with a fading of band intensity over the investigated time.

Tent cadavers also showed similar degradation patterns of vinculin and alpha actinin compared to open field pigs (Fig. 4D + E). The native forms of the proteins in tent pigs were fully degraded at 5 dpm for vinculin and within 5 to 10 dpm for alpha actinin (Fig. 5D + F). The meta-vinculin splice variant was fully degraded in all tent carcasses at 5 dpm (Fig. 5C), indicating a full degradation of the protein band within this time period. The vinculin split product of 63 kDa appeared in tent pigs from 5 dpm onwards (Fig. 5E). As in open-field pigs, tent carcasses also displayed degradation products of alpha actinin but showed no statistical regularity in their appearances.

## Discussion

The present data reveal that the morphological decomposition of the carcasses positioned in the open field was significantly faster than that of the carcasses lying in the tents. These findings are supported by a previous study which investigated the decomposition of pig cadavers encased in concrete compared to a non-encased control animal. The encased pigs also depicted a significantly slower decomposition rate compared to the control, and the PMI of the encased cadavers was underestimated [30]. Only after 1 year, the encased animals were in an early decomposition stage, whereas the non-encased pig already was already completely skeletonized.

The decomposition processes of the pig cadavers in the tents can be assumed to be decelerated due to several factors. It has repeatedly been shown that temperature is one of the most important external factors that influences the temporal dynamics of degradation. Thereby, decomposition rates of bodies always increase with increasing ambient temperatures [5]. This has also been shown for pigs, where high ambient temperatures together with direct sun exposure and increased humidity result in an accelerated decomposition process and that morphological changes appear in an advanced (chronological) manner [31]. It remains unclear whether the high humidity inside the tents negatively influenced the decomposition rate. Low humidity levels, in turn, often leads to mummification of corpses [32]. Decreased ventilation and restricted sun exposure may also be extrinsic factors that have the possibility to influence the putrefaction processes of (pig) cadavers [5]. A lack of oxygen exchange or water between cadavers and the surrounding environment can cause incomplete putrefaction of corpses [32] as observed in pig cadavers enclosed in tents.

A major factor, however, is the access of necrophagous organisms to the cadavers and fly oviposition. It is generally accepted that such insect activities accelerate the decomposition process [33, 34]. While in the open-field pigs a large abundance of fly larvae was observed on the carcasses very early and significant maggot masses were found on the cadavers throughout the experiment on different regions of the cadaver (mouth, eye, thoracic region, anal region, distal abdominal region), the access of necrophagous insects to the cadaver inside the tents was limited. In the latter, deposited eggs could be observed on the tent zippers as well as on the mesh window of the tents. This allowed limited access but still some insect colonization of the cadavers inside the tents; however, it was much later and much less abundant compared to open-field pigs. Particularly the early and abundant presence of blow flies appears to be an important factor as their activity has been proven to accelerate head decomposition and significant morphological changes [35]. All in all, the number of colonizing insects on tent cadavers differed significantly from openly placed carcasses. The limited insect activity on the tent pigs may explain the differences in the decomposition progress between the two treatment groups.

However, it is not only the necrophagous activity that increases the decomposition rate, it is also known that the maggot masses produce a considerably amount of heat which then again accelerates decomposition [34, 36]. This could also be observed in this field study where maggot masses on the cadavers caused an increase of the rectal temperatures in the first two days postmortem in all pigs. In addition, body temperatures of open-field pigs even displayed temperatures beyond ambient temperatures which can be attributed to extensive maggot colonization which led to an accelerated decomposition. These findings are also supported by another work based on this field study which particularly focuses on insect succession under different scenarios [37].

Another aspect that may influence morphological changes and may cause differences in the decomposition is the production and (lack of) draining of decomposition fluids. When a (vertebrate) body decomposes after death, there is a release of nutrient-rich fluids into the surface beneath, which can impact the soil and the associated compound structure and function. Studies show that certain peptides of these fluids are consistent with distinct PMI phases and correlate with the TBS of the cadaver and ambient temperature [38, 39]. It has also been shown that there are specific dynamics between cadavers and soil, especially the bacterial and arthropod community, as well as microbial function [40]. In a concealed environment such as tents, the draining of decomposition liquids is restricted, which cause accumulation of purged fluids around the cadaver, compared to other indoor scenarios like inside dwelling, or wrapped in carpets or blankets where bodies are commonly found dead [41]. In this field study, all tent pigs showed extensive accumulation of decomposition fluids. In comparison, soil drainage prevented purged fluids of open field pigs to accumulate under the cadavers and allowed mummification of the cadavers. The degradation of soft tissue essentially is a competition between putrefaction and desiccation which is influenced by several external as well as internal influences [42]. As previous studies have shown, the decomposition and mummification progresses much faster under outdoor conditions than indoors [43]. Since tents provided a closed environment with accumulated putrefactive fluids and relatively high humidity, this may explain, at least in parts, the difference in decomposition rates between the two treatment groups and the mummification of open field pigs compared to tent pigs.

All in all, evaluation of total body scores and postmortem morphological changes suggest that especially temperature and insect activity affect the decomposition process. Effects of air flow, exposure to sunlight, and draining of decomposition fluids may add to this difference but appear to be of minor importance.

Despite these differences in the morphology, postmortem muscle proteins showed similar degradation patterns, regardless of exposure. This confirms findings from previous studies which resulted in altered decomposition rates under sealed conditions [1]. The present field study could only confirm these results regarding postmortem morphological changes but suggests that postmortem protein degradation is mostly unaffected by exposure.

Muscle protein degradation patterns of open-field pigs largely align with those of tent pigs, which suggests that proteins degrade in a similar manner. Western blot results provide predictable and time-dependent patterns in early PMI phases (meta-vinculin, vinculin, alpha tubulin), but also and particularly in intermediate postmortem stages (vinculin degradation products, alpha actinin, GAPDH). In addition, this field study confirms findings from previous experiments that there are also proteins (tropomyosin) which are largely unaffected over long periods of time and remain stable in their native form without any signs of degradation [44].

However, again, temperature proves to be a major influencing factor for the proteolysis rate. In detail, higher ambient temperatures lead to accelerated degradation of muscle proteins compared to studies under lower temperatures [45, 46]. Since the present study took place during summer and under temperatures above average, it suggests that the protein method is applicable even under these conditions. Results of this field study also align with those of similar field studies and experiments implemented under controlled standardized environments [17, 19]. In detail, proteins like tropomyosin and vinculin depicted similar degradation patterns with either a stable double-banded native protein which is robust against environmental factors and postmortem degradation (tropomyosin), or a loss of the native protein band accompanied by several degradation products at distinct time points postmortem (vinculin) [44]. Similar to previous studies, alpha tubulin and GAPDH were characterized by a loss of the native band, and alpha actinin as well as vinculin additionally showed degradation products at distinct PMI phases [47].

In summary, results show that concealment of cadavers lead to different decomposition rates on a morphological level, but not on a molecular level, as protein degradation proved to be mostly unaffected. This is in accordance with previous studies, demonstrating that exposure of bodies only has partial effects on the protein degradation rates and that postmortem degradation patterns are mostly robust to variations in exposure [48]. Thus, the present field study on pig carcasses enables to create a model for protein degradation under natural environmental conditions and allows to predict degradation patterns that can be used for time since death estimates, regardless of whether cadavers decompose under natural conditions or in a mostly sealed microenvironment. In particular, the data reveal that protein degradation significantly correlates with PMI and certain influencing factors such as temperature and insect activity. Morphological evaluations, however, can only be used as an additional tool, due to the fact that morphological degradation is highly influenced by exposure. Nevertheless, present results should be validated by other field studies using human corpses due to some differences between species. For this purpose, taphonomic research outdoor facilities [49–51] become more and more important for such forensic investigations. Since there is currently only limited data regarding postmortem protein degradation in humans, this needs to be further focused on in future investigations.

## Key points

1. Five pig cadavers were left to decompose under natural environmental conditions (open field).
2. Five pigs were placed in tents in order to investigate postmortem decomposition under sealed conditions.
3. Postmortem morphological changes and postmortem protein degradation patterns were investigated.
4. Results show major differences in morphological changes postmortem between treatment groups (open field versus tents).
5. Postmortem protein degradation patterns of tent pigs are largely consistent with the open-field control pigs.

### Supplementary Information

Below is the link to the electronic supplementary material.

Fig. S1 Development of Accumulated Degree Days (ADD) of open field pigs (blue) and tent pigs [46], including and trend lines. Both treatment groups depicted the same trend and an increase of ADD values over the investigated time course of 10 dpm. ADD values of tent pigs are slightly higher than open field pigs. (TIF 56 KB)

File S1 Detailed description of muscle sample collection, sample processing, analysis via SDS-PAGE and Western blotting, and statistical analysis of all collected muscle samples of both treatment groups (open field and tent). (PDF 126 KB)

File S2 Detailed description of morphological changes and according Total Body Scores of all open field pigs over a time period of 25 days postmortem, and of tent pigs over a time period of 45 dpm. (PDF 290 KB)

## Data Availability

All data generated or analyzed during this study are included in the published article.

## Abbreviations

dpm: Days postmortem
GAPDH: Glyceraldehyde-3-phosphate dehydrogenase
PMI: Postmortem interval
ADD: Accumulated degree days
