# Supplementary figures and images for: Morphological changes and protein degradation during the decomposition process of pig cadavers placed outdoors or in tents—a pilot study
Source: Forensic Sci Med Pathol. 2023 May 1;20(2):508–17. doi: 10.1007/s12024-023-00632-3 (PMC11297119; doi:10.1007/s12024-023-00632-3)

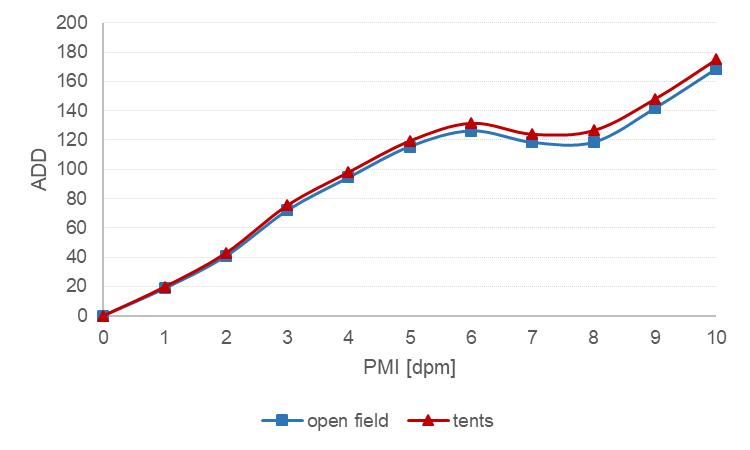

Supplement: Supplementary file 1 — Fig. S1 Development of Accumulated Degree Days (ADD) of open field pigs (blue) and tent pigs [46], including and trend lines. Both treatment groups depicted the same trend and an increase of ADD values over the investigated time course of 10 dpm. ADD values of tent pigs are slightly higher than open field pigs. (TIF 56 KB) [file 12024_2023_632_MOESM1_ESM.tif]
