# Supplementary material for: Morphological changes and protein degradation during the decomposition process of pig cadavers placed outdoors or in tents—a pilot study
Source: Forensic Sci Med Pathol. 2023 May 1;20(2):508–17. doi: 10.1007/s12024-023-00632-3 (PMC11297119; doi:10.1007/s12024-023-00632-3)
Supplement: Supplementary file 2 — File S1 Detailed description of muscle sample collection, sample processing, analysis via SDS-PAGE and Western blotting, and statistical analysis of all collected muscle samples of both treatment groups (open field and tent). (PDF 126 KB) [file 12024_2023_632_MOESM2_ESM.pdf]

Muscle sampling

Muscle samples were collected daily from each open field pig (n = 5) and every five days for each pig placed in a tent (n = 5). Samples from the *M. biceps femoris* were taken via biopsy and individually stored in 1 mL RIPA buffer (SIGMA) containing a protease inhibitor cocktail (ROCHE) in a cooled styrofoam box at the research outdoor station and later at -20 °C until further use, according to our established standard protocol with some adaptions [28]. The lysis and extraction buffer was used in order to prevent further muscle protein degradation after sample collection. For each sampling, a small incision (approximately 5 mm) was made through the skin and the underlying fascial layer of the cadaver using a surgical scalpel. From each pig, muscle tissue (approx. 5 × 5 × 5 mm) was extracted with a 5 mm biopsy needle from 2 cm depth within the hind limb. After sampling, incision wounds were sealed with cyanoacrylate glue (LOCTITE®), to avoid additional entry sites for insects and/or bacteria. In order to prevent interferences between sampling sites, a minimum distance of 2 cm was kept between each incision of subsequent samplings.

Sample processing

Back in the lab, homogenization of all obtained muscle samples was performed via a two-step process. After thawing of the frozen samples, the muscle tissue was dispersed using an *Ultra-Turrax®*. Homogenized samples were then further broken down by subsequent sonication via an ultrasonic processor (2 × 100 Ws/sample; *Vial Tweeter*) and centrifuged at 1000×*g* for 10 minutes. The supernatant was transferred into a new vial tube and stored at -20 °C until further use. The remaining pellets were discarded. Respective total protein concentration of all supernatants was individually measured using Pierce BCA-Assay Kit (Thermo Fisher Scientific Inc.) prior to sample analysis via SDS-PAGE and Western blotting.

SDS-PAGE and Western blotting

In a first step, all samples from each individual animal were diluted with double distilled water to a specific overall protein content (30 µg for vinculin, alpha-tubulin, α-actinin and GAPDH, 15 µg for tropomyosin) and a 25% sample buffer added (3 mM Tris pH 6.75, 8 % SDS, 40 % glycerol, 20 % mercaptoethanol, 0.05 % bromphenol blue) . Sample dilutions were denatured at 90 °C for 5 min prior to insertion into the stacking gel wells. Electrophoresis was performed according to Laemmli with some adaptions [29]. Proteins run on 10 % polyacrylamide resolving gels (acrylamide/N,N'-bismethylene acrylamide = 37.5:1, 0.1 % SDS, 0.05 % TEMED, 0.05 % APS, 375 mM Tris HCl, pH 8.8) and 5 % stacking gels (acrylamide/N,N'-bismethylene acrylamide = 37.5:1, 0.1 % SDS, 0.125 % TEMED, 0.075 % APS, 125 mM Tris HCl, pH 6.8) in an amount proportional to their molecular weight. Electrophoresis was run at a constant voltage of 150 V until the dye front reached the bottom of the resolving gel (approximately 2 hours). The running buffer contained 25 mM Tris pH 8.3, 195 mM glycine, 2 mM EDTA, and 0.1 % SDS. A protein ladder - a mix of dyed proteins with known molecular weight - was used to determine the size of the proteins of interest. Once the run was finished, the proteins were

transferred from the gels onto polyvinylidene fluoride (PVDF) membranes via electroblotting (wet transfer). For that purpose, a transfer buffer was used, containing 192 mM glycine, 20 % methanol, and 25 mM Tris pH 8.3. Electroblotting was run at a constant current of 250 mA for 75 min. Membranes were then stored at -20 °C until further use.

All membranes were blocked for 1 h in a blocking buffer containing PBST (137 mM NaCl, 10 mM Na_2HPO_4 anhydrous, 2.7 mM KCl, 1.8 mM KH_2PO_4, 0.05 % Tween) and 1 % bovine serum albumin as blocking agent (BSA; Albumin bovine Fraction V, pH 7.0). The following primary antisera were used for protein analysis of each pig: mouse monoclonal anti-vinculin (7F9, Santa Cruz Biotechnology, 1:1000), mouse monoclonal anti-α-actinin (H-2, Santa Cruz Biotechnology, 1:1000), mouse monoclonal anti-tropomyosin (CH1-s, DSHB, 1:500), mouse monoclonal anti-α-tubulin (12G10, DSHB, 1:500), mouse monoclonal anti-GAPDH (6C5, Santa Cruz Biotechnology, 1:1500). These antibodies were selected as suitable subjects due to existing data about their degradation behavior from previous studies. HRP-conjugated polyclonal goat anti-mouse immunoglobulins (Dako, 1:10·000) were applied as secondary antibodies. All primary and secondary antibodies were diluted in blocking buffer and incubated for 1 h each. After each antibody application, membranes were extensively rinsed and washed (3 × 10 min) in PBST. Visualization of antibody binding was enabled by application of the chemiluminescence substrate *SuperSignal West Pico PLUS* (Thermo Scientific™) and photographed using a digital gel documentation system *iBright CL1000* (Thermo Fisher™).

Statistical analysis

The intensity of all protein bands of each Western blot result was measured using the gel analysis tool of ImageJ software (v.1.48 NIH, National Institutes of Health, USA). Histograms of the tonal distribution of the images were plotted and the area underneath the graphs was measured according to the program's standard protocol. Band patterns of the 0 dpm samples were used as control and considered the native form of the protein on each blot. All band signals on the blot with ≥ 1 % relative density (compared to respective dominant control band) were considered a present protein band, all signals < 1 % of the respective control band were considered background. This enabled a binarization of the results and provided information on the absence (0) or presence (1) of proteins and degradation products. Means and standard deviations of the measured band intensities of each treatment group were assessed as a function of postmortem time of the native protein band using MS Excel. This allowed assessing, whether means and standard deviations of the two groups (open field versus tent) differed from each other, or if fluctuations were within random data distribution.
