# Supplementary material for: Morphological changes and protein degradation during the decomposition process of pig cadavers placed outdoors or in tents—a pilot study
Source: Forensic Sci Med Pathol. 2023 May 1;20(2):508–17. doi: 10.1007/s12024-023-00632-3 (PMC11297119; doi:10.1007/s12024-023-00632-3)
Supplement: Supplementary file 3 — File S2 Detailed description of morphological changes and according Total Body Scores of all open field pigs over a time period of 25 days postmortem, and of tent pigs over a time period of 45 dpm. (PDF 290 KB) [file 12024_2023_632_MOESM3_ESM.pdf]

Open field pigs

Due to the high ambient temperatures, the cadavers decomposed within only a few days and reached advanced decomposition stages very quickly. Total body scores developed from 3 at 0 dpm up to 29 within 7 days and to a maximum of 32 within 20 dpm (Fig.2).

In detail, on day 1 of the experiment (**0 dpm**), all pigs were in a fresh state (TBS = 3). Shortly after death (approx. 2 hours), adult flies started oviposition on the cadavers.

At **1 dpm**, four out of five pigs showed signs of early bloating and slight green discoloration of the ventral side of the trunk, corresponding to an early stage of decomposition. Additionally, neck and head displayed initiated bloating and red discoloration (TBS = 4-6). The fifth pig was still in a fresh state.

After **2 dpm**, four out of the five pigs showed extreme bloating of trunk, head and neck, partly accompanied with a dark green discoloration at the eye and the abdominal region. Fly maggot colonization was observed in the mouth (TBS = 5-7). One pig was in a less advanced decomposition stage where there was only a prominent green discoloration and bloating of the ear detectable (TBS = 5).

At **3 dpm**, the main characteristics of decomposition were a prominent bloating of head and neck with maggot and adult beetle colonization in mouth and eyes, as well as green and purple-black discoloration and marbling of abdomen with maximum bloat (partly accompanied with blisters at the ventral side of the trunk). At this advanced stage of decay, an initiated bloating including a prominent green discoloration of the extremities was visible (TBS = 10-11). The less advanced pig (TBS = 8) showed maggot colonization in the mouth, a slight green discoloration and marbling of abdomen and a bloating of proximal limbs.

At **4 dpm** (TBS = 14-15), head and neck of four cadavers were caved in due to extensive maggot infestation. Trunks were in a postbloated stage with gas release, dried blisters and partly extensive skin slippage at distal abdominal parts. Limbs showed dark green discoloration and marbling, partly accompanied by extensive skin slippage. In comparison, the less advanced pig displayed a black discoloration and extensive maggot colonization, as well as bone exposure of the jaw and dried lips and nose. The distal trunk showed skin slippage and blisters. Limbs appeared dry and discolored.

24 hours later (**5 dpm**), four carcasses showed a moist decomposition of head and neck with an exposed jaw bone and extensive maggot colonization. The trunk displayed dried brown areas with skin slippage, extensive hair loss, and partially remaining abdominal gas release. Limbs had a leathery appearance with extensive skin slippage. The outlying fifth pig had still a fleshy neck colonized by maggots. The trunk showed dry skin and skin slippage, especially at the distal end of the abdomen. The limbs displayed a controversial morphological appearance – proximal limbs were bloated, whereas distal extremities had a leathery appearance. TBS values were between 17 and 18.

At **6 dpm**, there was a complete bone exposure of the head including the jaw, and decomposed neck tissue detectable in four pigs (TBS = 23-26). This stage of decay was mainly characterized by extensive maggot colonization all over the cadaver. In addition, a beginning mummification of trunk and limbs

was visible, including leathery appearance and bone exposure in parts, especially at the dorsal side of the trunk. The less advanced pig (TBS = 17) showed partial bone exposure of head and jaw, and an extensive abdominal skin slippage with dried blisters. Proximal limbs were bloated whereas distal extremities already showed a leathery appearance including skin slippage.

On the next day (**7 dpm**), most of the maggots migrated away from the cadavers. Four carcasses showed colonizing beetle larvae and foam on trunk and limbs, as well as a caved in abdominal cavity with bone exposure of the rib cage (TBS = 25-26). The fifth pig displayed mummification of neck and bone exposure of the jaw (not the complete head), as well as a moist decomposition and partial bone exposure of the limbs (TBS = 22).

At **8 dpm**, the TBS values between 26 and 29 were reached amongst all cadavers. The differences in the scoring resulted mainly from the extent of bone exposure in the individual carcasses. Some cadavers already displayed exposed bone in the abdominal region (mainly rib cage), whereas others only showed bone exposure of skull and limbs.

These values only changed slightly over the following days. At **20 dpm**, the highest TBS value with 32 points were noted during this field study. (Fig.3)

<u>Tent pigs</u>

In general, the decomposition process of the pig cadavers placed inside tents was much slower than in the open field pigs. All pigs showed similar morphological changes at distinct PMI phases with one exception. This animal displayed an advanced decomposition rate compared to the remaining cadavers. Total body scores increased from 3 at 0 dpm up to a maximum of 29 within 45 dpm (Fig.2).

At the beginning of the experiment (**0 dpm**), all pigs were in a fresh state (TBS = 3).

At **5 dpm**, tents were opened for the first time and revealed similar initial morphological changes amongst all cadavers. Head and neck showed green discoloration and initial bloating with maggot colonization (mainly in the mouth region). Trunks and limbs were heavily bloated, partly accompanied with slight green discoloration. In addition, partly green marbling of the limb skin was detectable (TBS = 9-10).

At **10 dpm**, four pigs displayed a similar putrefaction progress with prominent head and neck bloating including black discoloration and drying of the nose. Post-bloated trunks showed black discoloration, purging of decomposition fluids, skin slippage and partly maggot-filled blisters. Limbs were bloated and showed green discoloration, partly accompanied with skin slippage (TBS = 11-12). Note that there was an extensive oviposition by adult flies detectable, both inside and outside the mesh of the tent. One of the cadavers displayed exceptional morphological changes and an advanced decomposition phase (TBS = 19). These changes included extensive skin slippage on neck and limbs, maggot colonization and migration of post feeding larvae to the tent corners, moist decomposition with foam building around the cadaver and bone exposure of trunk and limbs.

Five days later (**15 dpm**), total body values varied considerably amongst carcasses (TBS = 15-21). The most prominent morphological changes were decomposition fluids with considerable foam building around the cadavers, dry and firm-looking skin, extensive skin slippage, partial bone exposure of head and jaw. In one pig no organs remained and the whole cadaver looked viscous/liquefied.

At **20 dpm**, no major changes were detectable (TBS = 19-23). It has to be noted that at this point, total body scores of all tent cadaver were very similar within the treatment group.

At **25 dpm** and **30 dpm**, less maggot activity and moist decomposition with foam formation was detectable in four of the five tent cadavers. The exceptional pig showed mummification of the remaining skin of the limbs and only few maggots feeding on the cadaver (TBS = 21-26).

On the last observation day (at **45 dpm**), many fly pupae as well as beetles were observed, as well as heavy mold formation all over and around the cadavers. The exceptional pig additionally showed black coloring of exposed bones (TBS = 23-29). (Fig.3)
